# Supplementary figures and images for: Predicting functional networks from region connectivity profiles in task-based versus resting-state fMRI data
Source: PLoS One. 2018 Nov 12;13(11):e0207385. doi: 10.1371/journal.pone.0207385 (PMC6231684; doi:10.1371/journal.pone.0207385)

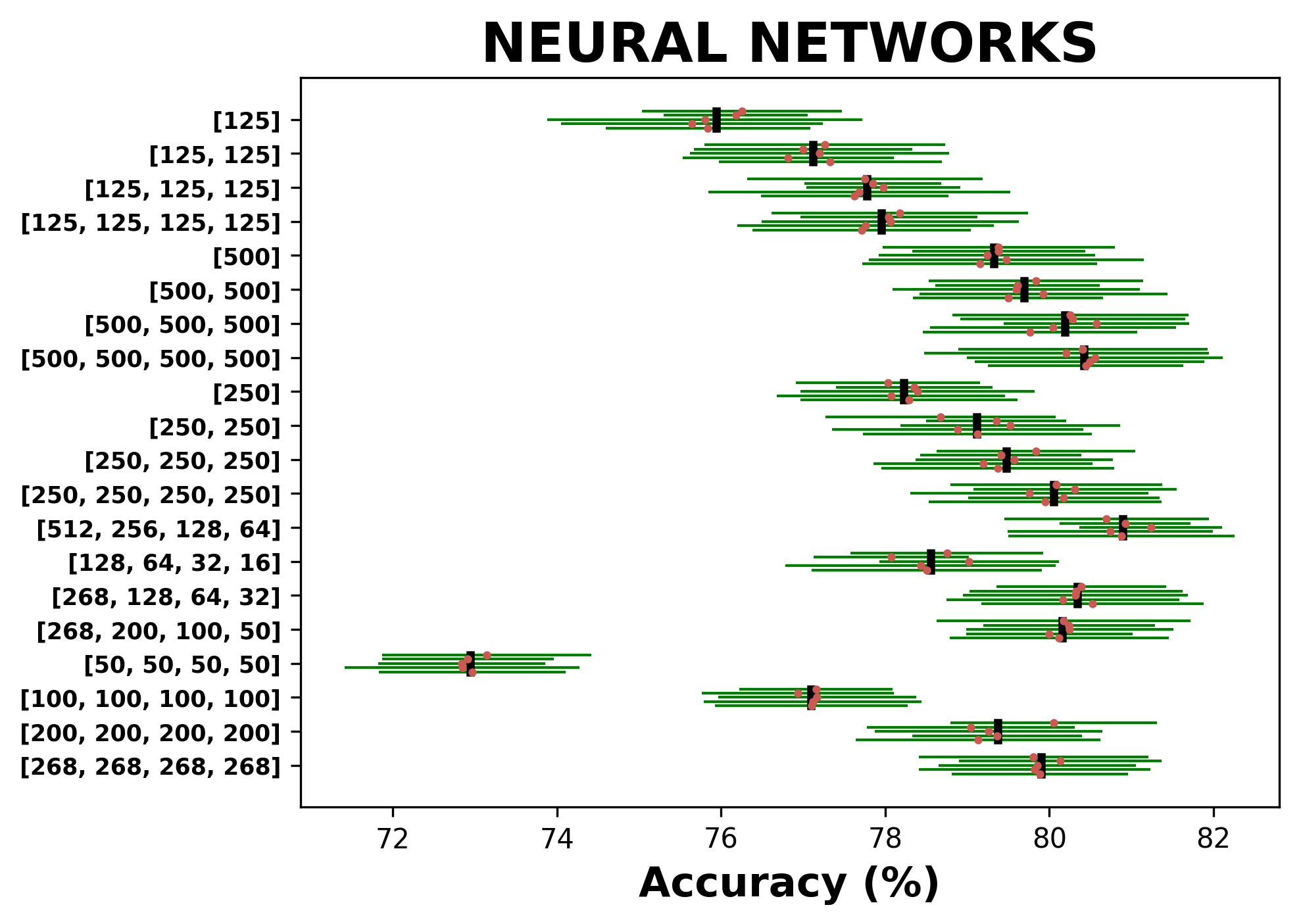

Supplement: S1 Fig — Global accuracy of a neural network with different layers (the number of components of each vector on the y-axis) and hidden units (the number within each component of the vectors on the y-axis) using a 5 times repeated 10-Fold Cross-validation exclusively on task fMRI. (TIFF) [file pone.0207385.s001.tiff]

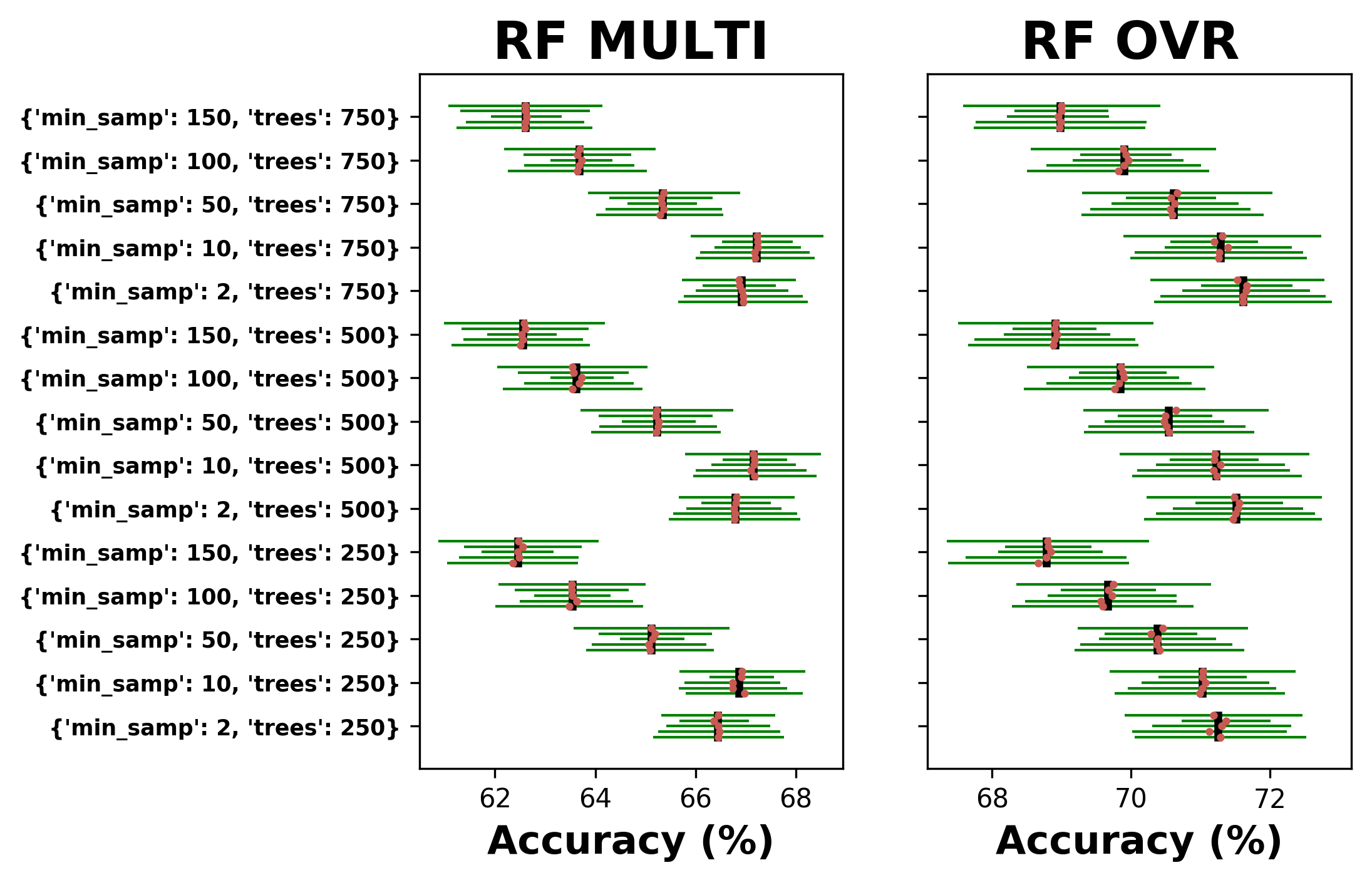

Supplement: S2 Fig — Global accuracy of a Random Forest, both in its multi-class and one-versus-rest version, varying the number of trees in the ensemble(“trees” parameter on the y-axis) and the minimum of sample in order to stop dividing each internal node (“min_samp” parameter on the y-axis) using a 5 times repeated 10-Fold Cross-validation exclusively on task fMRI. (TIFF) [file pone.0207385.s002.tiff]

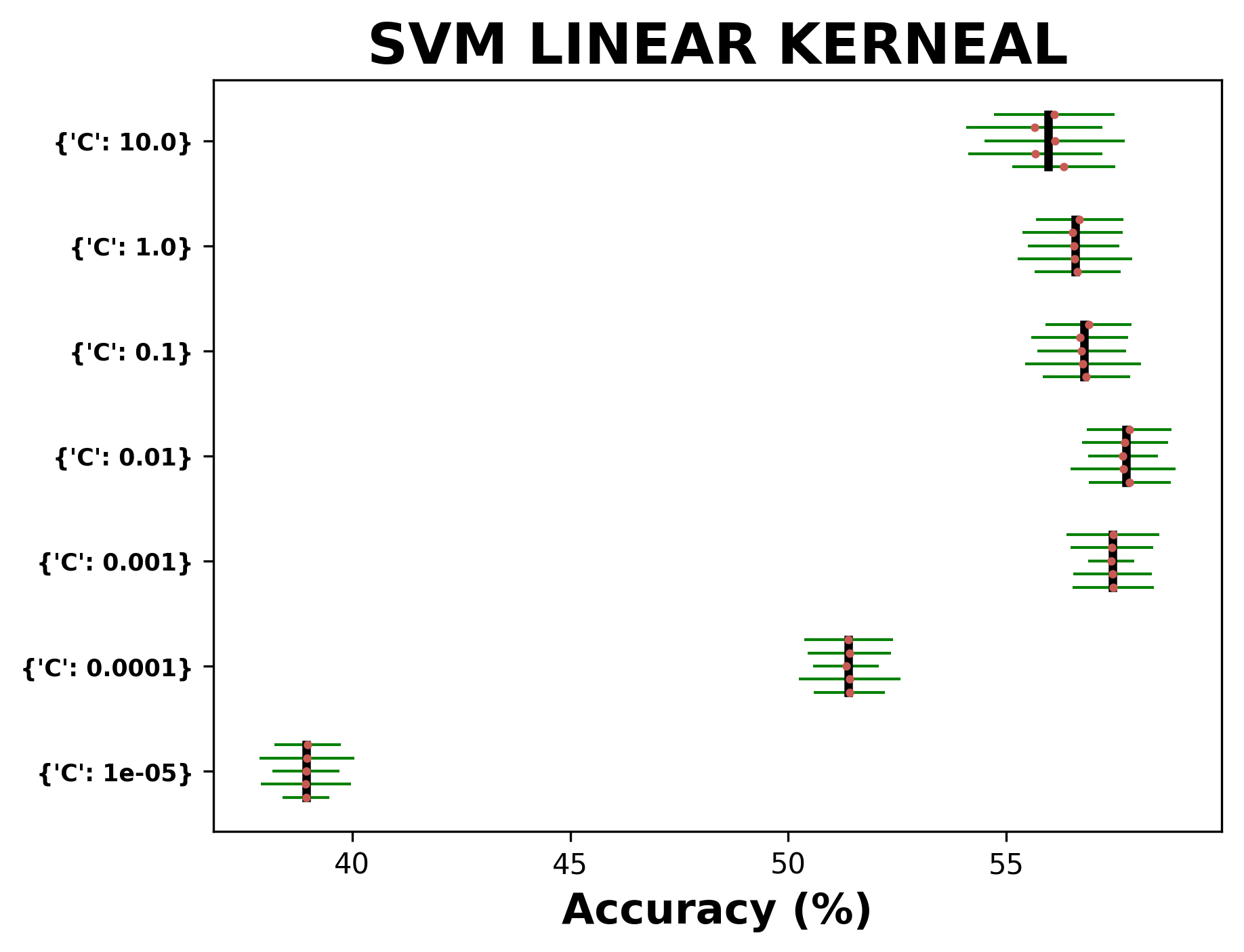

Supplement: S3 Fig — Global accuracy of a Support Vector Machine with a linear kernel varying the regularization coefficient (“C” parameter on the y-axis) using a 5 times repeated 10-Fold Cross-validation exclusively on task fMRI. (TIFF) [file pone.0207385.s003.tiff]

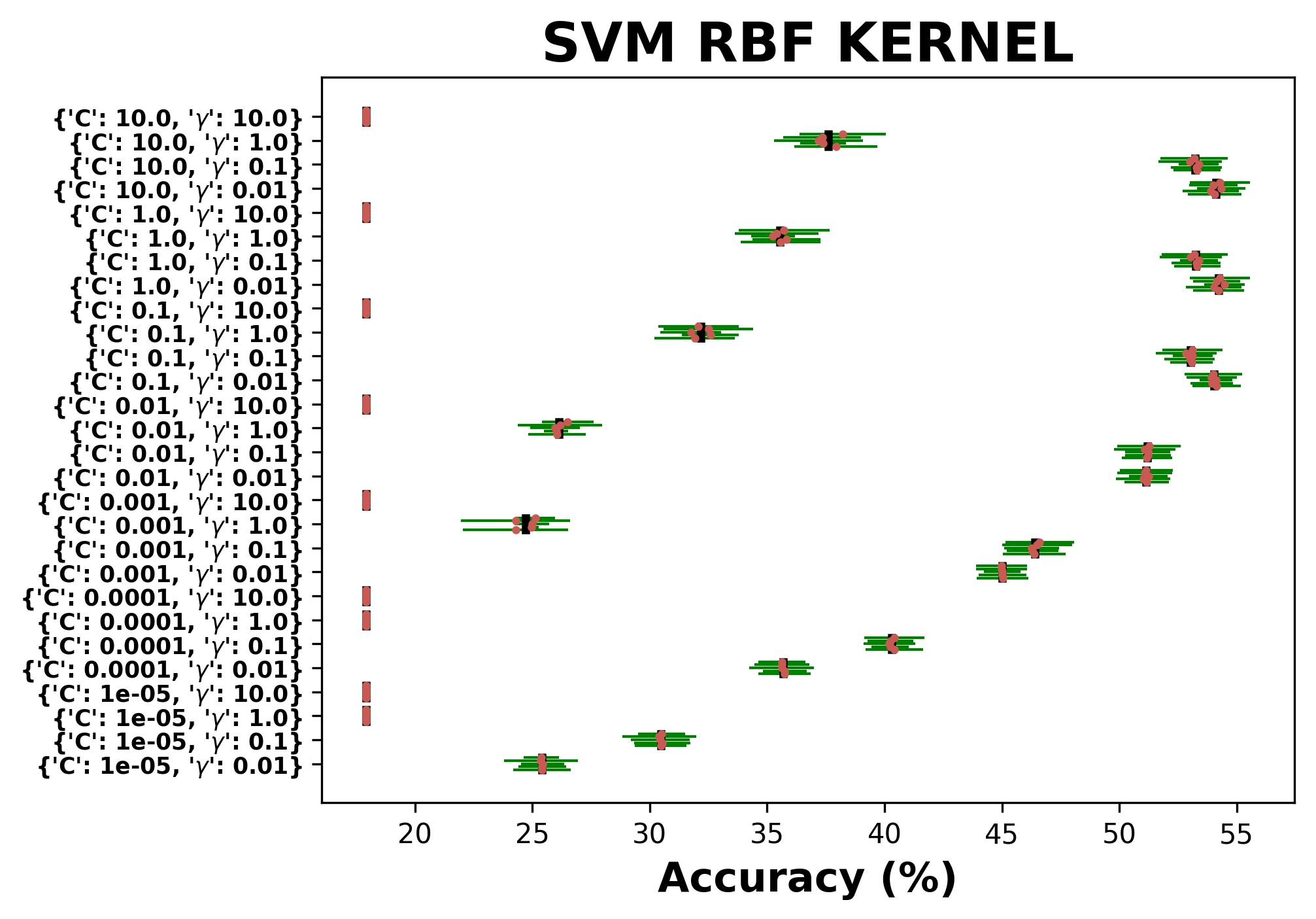

Supplement: S4 Fig — Global accuracy of a Support Vector Machine with a Radial Basis Kernel approximation varying the regularization coefficient (“C” parameter on the y-axis) and gamma kernel coefficient (γ on the y-axis) using a 5 Times repeated 10-Fold Cross-validation exclusively on task fMRI. (TIFF) [file pone.0207385.s004.tiff]
